# Characterizing avian pathogenic *Escherichia coli* (APEC) from colibacillosis cases, 2018

**DOI:** 10.7717/peerj.11025

**Published:** 2021-03-04

**Authors:** Darby M. Newman, Nicolle L. Barbieri, Aline L. de Oliveira, Dajour Willis, Lisa K. Nolan, Catherine M. Logue

**Affiliations:** 1 Department of Population Health, College of Veterinary Medicine, University of Georgia, Athens, GA, USA; 2 Department of Infectious Diseases, College of Veterinary Medicine, University of Georgia, Athens, GA, USA

**Keywords:** *Escherichia coli*, APEC, Characterization, Colibacillosis, Diagnostic, Case

## Abstract

Colibacillosis caused by avian pathogenic *Escherichia coli* (APEC) is a devastating disease of poultry that results in multi-million-dollar losses annually to the poultry industry. Disease syndromes associated with APEC includes colisepticemia, cellulitis, air sac disease, peritonitis, salpingitis, omphalitis, and osteomyelitis among others. A total of 61 APEC isolates collected during the Fall of 2018 (Aug–Dec) from submitted diagnostic cases of poultry diagnosed with colibacillosis were assessed for the presence of 44 virulence-associated genes, 24 antimicrobial resistance genes and 17 plasmid replicon types. Each isolate was also screened for its ability to form biofilm using the crystal violet assay and antimicrobial susceptibility to 14 antimicrobials using the NARMS panel. Overall, the prevalence of virulence genes ranged from 1.6% to >90% with almost all strains harboring genes that are associated with the ColV plasmid—the defining trait of the APEC pathotype. Overall, 58 strains were able to form biofilms and only three strains formed negligible biofilms. Forty isolates displayed resistance to antimicrobials of the NARMS panel ranging from one to nine agents. This study highlights that current APEC causing disease in poultry possess virulence and resistance traits and form biofilms which could potentially lead to challenges in colibacillosis control.

## Introduction

*Escherichia coli* is a Gram-negative bacterium member of the family *Enterobacteriaceae* that commonly inhabits the intestines of many warm-blooded organisms. In poultry, disease caused by extra-intestinal associated *E. coli* (APEC) is a significant cause of economic loss. APEC have been implicated in disease of production birds including broilers, layers, turkeys, geese, etc. resulting in clinical syndromes that include cellulitis, omphalitis, colibacillosis, airsacculitis, and salpingitis among others (Barbieri et al., 2015; Dias da Silveira et al., 2002; Maluta et al., 2016; Nakamura et al., 1997; Nolan et al., 2013). Infection associated with APEC is often viewed as secondary to other predisposing factors such as compromised immune system or mucosal and skin barriers that can no longer provide protection against the pathogen (Nolan et al., 2015, 2013), however research by our lab and others has demonstrated that APEC can also be a primary pathogen (Collingwood et al., 2014; Nolan et al., 2013). In addition, vertical transmission of APEC has been reported from infected hens to progeny (Nolan et al., 2015).

Several APEC virulence factors are recognized; however no single virulence trait has been found to identify an APEC as an APEC. Recognized virulence traits of APEC include factors such as adhesins, toxins, iron acquisition mechanisms, invasins, and plasmids (Nolan et al., 2015, 2013, 2020). The type 1 fimbriae (*fim*), temperature sensitive haemagglutinin (*tsh*), iron-scavenging systems including *iroN, feoB, chuA, fyuA, ireA, irp2, iucD and sitD*, increased serum survival gene (*iss*) and protectins are also included (Barbieri et al., 2013; Johnson et al., 2007a, 2012a, 2006a, 2008a, 2012b; Rodriguez-Siek et al., 2005). In addition, some of these genes are linked with APEC plasmids such as Colicin V (ColV) plasmids (Barbieri et al., 2013; Johnson et al., 2006a) which are considered a defining trait of the APEC pathotype and are significantly associated with disease-causing APEC (Johnson et al., 2012a, 2006a; Nolan et al., 2013; Rodriguez-Siek et al., 2005; Skyberg et al., 2006). The bacterium does not, however, need to possess all of these virulence factors to be pathogenic and APEC often use a “mix and match” approach to cause disease (Dobrindt, 2005).

Biofilms occur in a variety of environments and can be detrimental in the healthcare and poultry production industry alike, they consist of a complex, three-dimensional, surface associated, polysaccharide protective coat around a community of cells (Donlan & Costerton, 2002; Flemming & Wingender, 2010). In biofilms, genes and plasmids can be taken up by members of the biofilm to enhance survival, increase genetic diversity and promote resistance to antibiotics or other sanitizing agents (Beloin, Roux & Ghigo, 2008; Davey & O’Toole, 2000). Under stressful conditions such as nutrient starvation, antibiotic presence or host immunological defenses biofilm formation can occur (Beloin, Roux & Ghigo, 2008). In human medicine, persistent and chronic infections are often linked with biofilms with an estimated 65% of hospital infections having a biofilm origin (Potera, 1999). Similarly, biofilms are a significant cause of concern for producers and the food processing industries alike as it can lead to problems of fouling, contamination of supply lines such as waterers/drinkers (Maes et al., 2019) and on process lines cross contamination of foods, potentially posing a health risk for consumers (Galie et al., 2018; Lindsay, Geornaras & Von Holy, 1996). In APEC, biofilm formation has been described by a number of researchers, including our group, who reported different biofilm formation types ranging from negligible and weak to strong that was linked to media type used (Nielsen et al., 2018; Skyberg et al., 2007).

Subtyping of *E. coli*, in particular extra-intestinal strains, relies on the use of phylogenetic typing which was first described by Clermont, Bonacorsi & Bingen (2000). The scheme was built on a typing scheme first proposed by Herzer et al. (1990) and Selander, Caugant & Whittam (1987) and uses a simple triplex PCR consisting of two genes (*chuA*, *yjaA*) and an anonymous DNA fragment (TSPE4.C2) (Gordon et al., 2008) resulting in four types A, B1, B2 and D. Good correlation was found between phylogenetic typing and other subtyping methods such as multi locus sequence typing (MLST) (Gordon et al., 2008). Updates to the scheme has expanded the phylogenetic types to include A, B1, B2, C, D, E and F and additional clades for cryptic strains (Clermont et al., 2013). Phylogenetic typing has worked well for human strains with most disease causing ExPEC classifying as phylogenetic group B2 and to a lesser extent phylogenetic group D while commensal human strains were assigned as group A (Clermont, Bonacorsi & Bingen, 2000; Picard et al., 1999). Application of phylogenetic typing to another ExPEC—APEC, found that not all pathogenic avian strains classified as B2 and are more likely to classify as phylogenetic groups B1, C and F (Rodriguez-Siek et al., 2005). In addition, use of the revised 2013 phylogenetic typing scheme (Clermont et al., 2013) resulted in re-classification of some APEC isolates to phylogenetic types C, E and F suggesting that these phylogenetic types also represent pathogenic strain types and may be influenced by pathogenicity islands they harbor (Logue et al., 2017).

Antimicrobial resistance and virulence in APEC is well known and often related to mobile genetic elements such as plasmids and transposons (Johnson et al., 2002, 2010a, 2005, 2006a, 2010b, 2006b; Johnson, Johnson & Nolan, 2006; Kariyawasam et al., 2006). Plasmid replicon typing is one such method for characterizing plasmids associated with virulence and resistance. The original replicon typing schemes were developed by Carattoli (2009) and Carattoli et al. (2005) and later modified by Johnson & Nolan (2009). Studies of plasmid replicons associated with pathogenic and commensal *E. coli* of poultry have demonstrated a diversity of plasmids among examined strains (Johnson et al., 2012a, 2007b). Our research group and others have highlighted the association between pathogenic APEC of production birds and the presence of the ColV plasmid which is a defining trait of the APEC pathotype (Johnson et al., 2006a, 2008a).

Also of interest in this study is an assessment of the antimicrobial susceptibility of APEC to agents of the national antimicrobial resistance monitoring system (NARMS) panel that focuses on antimicrobials of concern for both human and animal health. In an era of changes to the use of antimicrobials in animal production as a consequence of the veterinary feed directive (VFD) (Food and Drug Administration, 2015) and consumer pressure for an antimicrobial-free product, data on the impact of these changes on pathogens such as APEC is currently limited.

A variety of APEC serogroups have been identified among APEC examined to date including some of the more common serogroups such as O1, O2, O21, O35, O36 and O78 (Dziva & Stevens, 2008; Fulton & Boulianne, 2013; Knobl et al., 2012; Rodriguez-Siek et al., 2005). However, these serogroups only represent a fraction of the APEC serogroups that have been implicated in disease which poses a significant challenge for vaccine development as no single vaccine is broadly protective against the diversity of APEC strains causing disease (Lynne et al., 2012).

The purpose of this study was to characterize APEC strains isolated from lesions of colibacillosis in broilers and to evaluate their virulence and resistance gene possession, antimicrobial susceptibility and biofilm formation and to assess for potential correlations.

## Materials and Methods

### Samples and hemolysis reaction

All APEC isolates used in this study were recovered from tissues of poultry that were diagnosed with colibacillosis (see Table 1; Table S1). Isolates were recovered from different lesions and locations including bone marrow, yolk sac, ovary, lung, air sac, brain, peritoneum, heart, and liver. *E. coli* isolates (de-identified) were received (*n* = 61) on tryptone soy agar (TSA) plates with 10% sheep blood following incubation at 37 °C for 18–24 h. All plates were inspected for hemolysis (Rodriguez-Siek et al., 2005) and results recorded. Isolates included in this study were collected between August and December 2018.

**Table 1:** APEC isolates examined in this study including tissue source, serogroup, phylogenetic group and plasmid replicon types.

Study ID	Tissue source	Serogroup	Phylogenetic group	Replicons
1	Bone marrow	25	B2	
2	Yolk sac	86	E	
3	Yolk sac	86	E	I1
4	Yolk sac	86	E	
5	Yolk sac	18	E	
6	Peritoneum	24	A	K/B, FIB
7	Yolk sac	86	D	
8	Yolk sac	2	B2	
9	Ovary	78	B2	
10	Liver	78	B2	
11	Bone marrow	78	B2	I1
12	Bone marrow	86	D	
13	Ovary	24	F	
14	Bone marrow	24	F	
15	Bone marrow	24	F	
16	Ovary	2	B2	
17	Ovary	A*	F	
18	Ovary	24	F	
19	Ovary	24	F	
20	Bone marrow	24	F	
21	Bone marrow	24	F	
22	Bone marrow	R**	F	
23	Bone marrow	24	F	
24	Lung	186	B1	I1
25	Lung	186	B1	I1
26	Lung	186	B1	I1
27	Air sac	17/73/77/106	E	
28	Air sac	17/73/77/106	E	FIB
29	Air sac	17/73/77/106	E	
30	Ovary	78	F	
31	Ovary	78	F	
32	Air sac	78	F	
33	Air sac	78	F	
34	Brain	78	C	I1
35	Pericardium	78	C	
36	Air sac	78	C	
37	Bone marrow	2	B2	
38	Pericardium	2	B2	
39	Liver	2	B2	
40	Lung	24	A	
41	Heart	19	D	FIB
42	Heart	19	D	FIB
43	Heart	19	D	FIB
44	Bone marrow	25	B2	I1
45	Liver	25	B2	I1
46	Liver	78	unknown	Y, I1
47	Egg	78	unknown	Y
48	Bone marrow	78	unknown	Y
49	Bone marrow	78	F	FIB
50	Egg	78	F	FIB
51	Liver	78	F	FIB
52	Bone marrow	25	B2	FIB, I1
53	Liver	25	B2	FIB, I1
54	Lung	25	B2	FIB, I1
55	Heart	20	unknown	FIB, I1
56	Body cavity	178	B1	FIA
57	Air sac	178	B1	FIIA
58	Heart	20	A	FIB
59	Body cavity	178	B1	FIA
60	Lungs	8	B1	
61	Lungs	2	B2	K/B

**Notes:**

* A indicates there was autoagglutination and a serotype could not be determined.

** R indicates a rough colony and a serotype could not be determined.

All isolates examined were lactose positive and demonstrated gamma hemolysis.

### Lactose fermentation

Isolates were struck to MacConkey (MAC) agar and incubated at 37 °C for 18–24 h and identified as lactose fermenters if the colonies and media turned pink (Rodriguez-Siek et al., 2005).

### Serogrouping

All isolates were submitted to the *Escherichia coli* reference center (Pennsylvania State University, University Park, PA) for serogrouping using slide agglutination.

### Antimicrobial susceptibility analysis

Antimicrobial susceptibility was assessed using the NARMS panel (CMV3AGNF; Sensititre; ThermoFisher, Waltham, MA, USA) according to clinical laboratory standards institute (CLSI) guidelines (CLSI, 2019). Isolates were grown on TSA at 37 °C for 18 h and colonies picked into 5 mL of sterile water and adjusted to a 0.5 McFarland, then 10 µL of the suspension was mixed with 11 mL of cation adjusted Mueller Hinton (MH) broth with TES and vortexed well to ensure even distribution of the cells. The cell suspension (50 µL) was distributed into each well of the 96 well NARMS panel using a Sensititre AIM system. The plates were sealed and incubated at 37 °C for 18 h. Minimum inhibitory concentrations of each antimicrobial were determined using the Sensititre Optiread system. Data for MICs and break points were determined using CLSI standards (M100) (CLSI, 2019) and strains were recorded as resistant to the antimicrobial if the MIC was at or above the recommended breakpoint.

### DNA extraction

A single colony from blood agar was streaked on MacConkey agar and grown at 37 °C for 24 h. An individual colony was taken from the MacConkey agar and inoculated into 1 mL of sterile Luria-Bertani (LB) broth and incubated at 37 °C for 24 h. After centrifugation (10,000×*g*) for 2 min, the supernatant was removed and 200 µL of sterile molecular grade water (LifeSciences; ThermoFisher, Waltham, MA, USA) added and the cells mixed. The cell suspension was boiled at 100 °C for 10 min on a heating block (Isotemp; Fisher Scientific, Dubuque, IA, USA) and then allowed to cool before centrifuging again to remove cellular debris and 150 µL of the DNA supernatant transferred to a new tube. The DNA samples were stored at −20 °C until use.

### 16S *Escherichia coli* confirmation

Isolates from MacConkey agar plates of typical morphology were identified as *E. coli* using a polymerase chain reaction (PCR) targeting the 16S DNA as described previously (Lamprecht et al., 2014). Amplification of the gene target was carried out as described below.

### PCR amplification

All PCR analysis for characterizing *E. coli* (16S, virulence traits, antimicrobial resistance traits, replicon types and phylogenetic grouping) was carried out using the following protocol with minor modifications for annealing temperatures of the primers (see Table S2). DNA samples were amplified using polymerase chain reaction (PCR) in multiplex panels to amplify a series of 44 virulence-associated genes in APEC (Johnson et al., 2008b; Logue et al., 2012). Phylogenetic typing was carried out as previously described (Clermont et al., 2013) which classifies *E. coli* into phylogenetic groups A, B1, B2, C, D, E and F; plasmid replicon typing which classifies plasmid replicons harbored by strains (plasmid types) (Johnson & Nolan, 2009; Johnson et al., 2007b); and some of the common antimicrobial resistance genes (Johnson et al., 2008b) harbored by strains.

All PCR reactions were prepared in a total volume of 25 µL for each sample. Components for a PCR reaction consisted of 2.5 µL of 10X PCR buffer, 1.25 µL of (0.2M) dNTP mixture, 0.4 µL of TAQ (Dream TAQ; ThermoFisher, Waltham, MA, USA), 1.4 µL of primer pool, 2 µL of DNA, and 17.45 µl of sterile molecular grade water. Positive control strains were included in the analysis for the appropriate genes of interest from previously characterized strains in our lab collection and negative controls included sterile water in place of DNA.

Amplification parameters of the thermocycler (Mastercycler X50; Eppendorf, Hamburg, Germany) included an initial denaturing step at 95 °C for 10 min, followed by 30 rounds of (94 °C for 30 s, (various annealing temperatures) for 30 s, 68 °C for 3 min), with a final extension of 72 °C for 10 min and a final hold of 4 °C.

Polymerase chain reaction products generated were subjected to electrophoresis which was performed in a 1.5% agarose gel (Agarose LE; Lonza, Alpharetta, GA, USA) running at 100V for 90 min. The gel was stained with ethidium bromide (0.25%) solution for 20 min, visualized using an imager (UVP BioDock-It^2^ Imager; Analytik Jena, Jena, Germany) and analyzed for the presence of PCR products of the appropriate size when compared with lab control strains for the targeted gene.

### Biofilm analysis

The procedure was followed from previous work on biofilms that used M63 minimal media (Nielsen et al., 2018; Skyberg et al., 2007). Test strains were streaked out on Luria–Bertani (LB) agar and incubated at 37 °C for 18–24 h. Then, single colonies were picked and transferred to 2 mL of LB broth. The samples were grown for 16 h at 37 °C. M63 media was prepared as described previously (Nielsen et al., 2018; Skyberg et al., 2007).

Next, 2 μL of inoculum was added to 198 μL of M63 minimal media in each well of a 96 well microtiter plate (Sarstedt, Germany). Eight wells were used for each test strain with a total of 11 isolates tested per plate; the 12th column of wells was used as a negative control (no growth) containing the uninoculated broth only. A sterile lid was placed over the plate (Sarstedt, Germany) and the plates were incubated at 37 °C for 24 h. After 24 h of growth, the broth in each well was poured off and the wells gently washed with sterile molecular grade water 3 times. Washing and staining procedure and biofilm re-suspension was carried out as previously described (Nielsen et al., 2018; Skyberg et al., 2007) with the density of the biofilm measured at OD 600 nm using an automated ELx808 Ultra Microplate Reader (Bio-Tek, Winooski, VT, USA). Biofilm density in each of the 8 wells for each test strain was averaged and compared to the OD of the negative control wells. Biofilm density was classified as negligible (no biofilm) had an OD lower than the OD of the control wells (i.e., OD < ODc), weak biofilms were classified as ODc < OD < (2 × ODc); moderate biofilms were classified as (2 × ODc) < OD < (4 × ODc) and strong biofilms were classified as (4 × ODc) < OD (Skyberg et al., 2007).

### Statistical analysis

Data was analyzed using non-parametric tests due to asymmetry in the distribution of genes or other traits used for analysis. In addition, this analysis excluded isolates from the brain, egg and body cavity/peritoneum due to the very limited number of isolates in each group (1–3 isolates).

Tukey’s multiple comparisons methods were used for comparisons among serogroup and tissue of isolation (Table S3A); for phylogenetic groups and tissue of isolation (Table S3B), and for biofilm and tissue of isolation (Table S3F).

For the analysis of virulence and resistance genes harbored by strains examined in the study, the number of genes was treated as the quantitative variable and the data was analyzed using non-parametric tests also due to asymmetry in the distribution of these genes. Direct comparisons (where possible) between two groups (Table S3D) were made using the Mann–Whitney U test.

The chi square test was used to allow comparison of phenotypical antimicrobial resistance, antimicrobial resistance genes, virulence genes, plasmid replicons, and biofilm strength (Table S3E).

A chi square test was used for the analysis of serogroup and phylogenetic group (Table S3C).

All statistical analysis was performed using GraphPad Prism (Version 7.0d) for MAC OS X (GraphPad, La Jolla, CA, USA) or IBM SPSS Statistics (Version 26.0) for MAC OS X (IBM Corp., Armonk, NY, USA). Statistical significance was accepted when *p* < 0.05.

## Results

Table 1 shows data on all *E. coli* isolates used in this study. All isolates displayed lactose fermentation (positive) on MAC and displayed gamma hemolysis on blood agar.

### Serogroups

**Figure 1:**
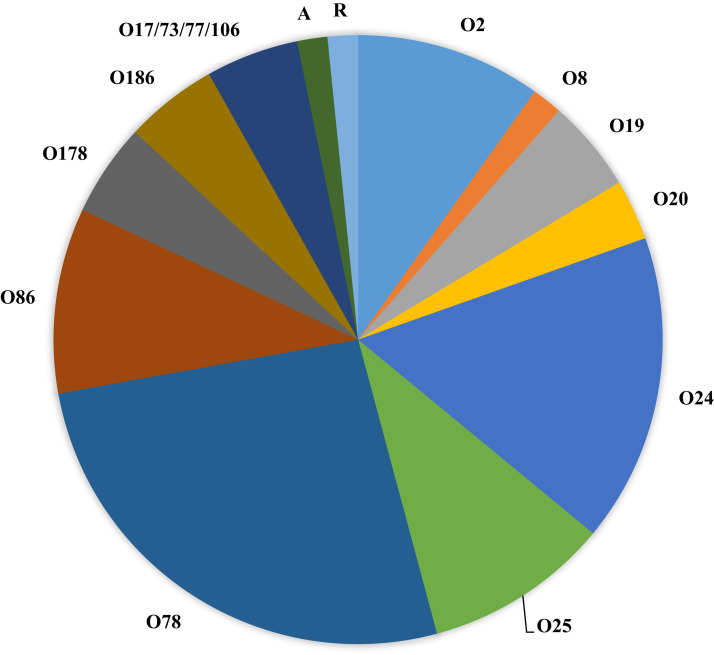
Serogroups detected among APEC isolates examined. Serogroups identified among the APEC collection examined using slide agglutination. Of interest, the collection was dominated by serogroups O2, O24, O25, O78 and O86. Of these, serogroups O2 and O78 are typically associated with APEC. A—autoagglutinated; R—rough colony.

Figure 1 (and Table 1) shows the serogroups detected among all APEC analyzed. Of interest, the dominant serogroups represented included O78 (26.2%), O24 (16.3%), followed by O2, O25 and O86 (9.8%). Two isolates either autoagglutinated or were rough and could not be serogrouped and 3 isolates presented with multiple serogroups (17/73/77/106).

Applying Tukey’s multiple comparisons test to compare tissue of isolation and serogroups (Table 3A) found no significant associations. However, when serogroups detected in specific organs were compared, significant associations were noted for serogroup O24 strains when compared for bone marrow vs heart; vs liver and vs yolk sac isolates (*p* < 0.05).

### Phylogenetic groups

Table 2 (and Table 1) shows the distribution of the phylogenetic groups identified among the APEC examined in this study. The majority of isolates were of phylogenetic groups B2 and F (24.5% and 27.8% respectively). Lower prevalence were observed for phylogenetic groups A, B1, C, D and E ranging from 4.9% to 11.4%. In addition, 4 isolates could not be typed using the current phylogenetic typing scheme and are designated as unknown and likely cryptic (Clermont et al., 2013). Table 2 also shows the assignment of all strains in each phylogenetic group and their ability to harbor genes associated with the APEC pentaplex (Johnson et al., 2008a), and the serogroups found among isolates in each phylogenetic group. Of interest, most strains were positive for the *hlyF*, *iroN*, *iss* and *ompT* (93.4–95%); however, the prevalence of *aerJ* was considerably lower at 46.5% prevalence while the prevalence of *iss* was 88.5%.

**Table 2:** Phylogenetic group distribution, virulence genes and serogroups identified among APEC examined. Phylogenetic distribution of APEC and their associated virulence gene profiles and serogroups.

Phylogroup	Prevalence (%)	*hlyF*	*iroN*	*iss*	*aerJ*	*ompTp*	Serogroups (No.)
A	3^a^ (9.6)^b^	3	2	3	2	2	O24 (2)^c^; O:20 (1)
B1	7 (11.4)	4	4	3	3	4	O186 (3); O:178 (3); O:8 (1)
B2	15 (24.5)	15	15	12	6	15	O:25 (6); O:2 (6); O:78 (3)
C	3 (4.9)	3	3	3	0	3	O:78 (3)
D	5 (8.1)	5	5	5	0	5	O:86 (2); O:19 (3)
E	7 (11.4)	7	7	7	3	7	O:18 (1); O:86 (3); 17/73/77/106 (3)
F	17 (27.8)	17	17	17	13	17	O:24 (8); O:78 (7); R (1); A (1)
Unknown	4 (6.5)	4	4	4	0	4	O:78 (3); O:20 (1)
Total	61	58 (95)^d^	57 (93.4)	54 (88.5)	27 (46.5)	57 (93.4)	

**Notes:**

a Number of isolates in each phylogenetic group.

b % of isolates in each phylogenetic group.

c Number of isolates in each serogroup.

d % prevalence in each group.

When comparative analysis between tissue of isolation and phylogenetic group was assessed using Tukey’s multiple comparisons test (Table 3B) no significant associations were observed (*p* > 0.05) for each individual phylogenetic group and tissue source. Similarly, when comparative analysis was carried out for individual tissue of isolation and phylogenetic groups detected among the isolates for each tissue type, no significant differences were observed (*p* > 0.05) either.

### Phylogenetic groups and serogroups

Comparative analysis of the relationship between phylogenetic grouping and serogrouping of all isolates was carried out using the chi-square analysis (Table S3C). A strong association (*p* < 0.05) was found between serogroup and phylogenetic group. However, these associations were not exclusive to one serogroup or phylogenetic group alone. As an example, phylogenetic B2 was significantly associated with serogroups O2 and O25; similarly, phylogenetic group E was significantly associated with O18, O86 and O17/73/77/106.

### Virulence genes

**Figure 2:**
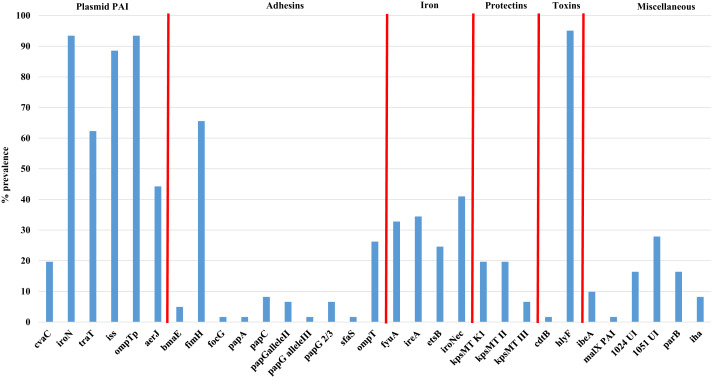
Prevalence of virulence genes among APEC examined. Virulence genes associated with ExPEC including plasmid associated pathogenicity island genes, adhesins, iron associated genes, protectins, toxins and a miscellaneous group of genes. Genes not detected in any isolate included *gafD*, *papEF*, *papG allele1*, *papG allele 1′*, papG*, *sfa-foc*, *adhC*, *cnf-1*, *hlyD*, *fliC (H7)*, *umuC*, *rfc*, and *afa*.

Figure 2 shows the overall prevalence of virulence genes detected in all 61 APEC examined. Genes such as *iroN, ompTp*, and *hlyF* were found to occur at the highest frequency in >90% or more of isolates examined while the prevalence of other virulence genes varied significantly from non-detection to 88% prevalence. Of significant interest was the prevalence of genes associated with the Col V plasmid which includes *aerJ, hlyF, iss, iroN*, and *ompTp* that were detected in 44.3, 95.0, 88.5, 93.4 and 93.4% of isolates respectively. The following genes were not detected in any isolate: *gafD*, *papEF*, *papG allele1*, *papG allele 1’*, papG*, *sfa-foc*, *adhC*, *cnf-1*, *hlyD*, *fliC (H7)*, *umuC*, *rfc*, and *afa*.

Using the Mann–Whitney U test (Table S3D), to compare virulence gene prevalence with tissues of isolation found some significant relationships (*p* < 0.05) between certain genes and the tissues they were affiliated with, including genes such as iron related *fyuA*, and *ireA* and lungs vs bone marrow and heart; the adhesin *fimH* and lungs vs liver; bone marrow vs liver; liver vs ovary and yolk sac and genes of the PAI including *aerJ* (bone marrow vs heart; heart vs ovary and yolk sac; and liver vs ovary) and *traT* (heart vs yolk sac, and liver; and liver vs ovary). Similarly, using the chi-square test (Table 2E), that allows comparison between the presence of two genes analyzed in all strains, significant associations were observed for some specific genes such as *fimH* and a number of other genes including *ireA*, *fyuA*, *aerJ*, *ompT*, *kpsII*, *traT*, *iss*, and *hlyF* (*p* < 0.05).

### Plasmid replicons

Analysis for the detection of plasmid replicon types among the APEC examined found that FIB (21.3%) and I1 (21.3%) were the most common plasmid replicon types detected. A total of 28 isolates harbored at least 1 plasmid replicon and 5 of those harbored a second replicon (see Table 1; Table S1). Other replicon types detected at low prevalence included K/B, FIA, FIIA and Y (<5% prevalence).

Analysis using the Mann–Whitney U test comparing replicon types and tissue of isolation (Table S3D) found significant associations between heart isolates and those of bone marrow, ovary, lungs and yolk sac (*p* < 0.05) for replicon type FIB only; no such associations were observed for any of the other replicon types. Similarly, using the chi-square test (Table S3E) significant associations were noted between plasmid replicon types and a range of genes and phenotypes including antimicrobial resistance (phenotype) (I1 vs AUG, AMP, FOX, XNL, and AXO); FIA vs AMP, FIB vs FIS; Y vs NAL and TET antimicrobial resistance genes (FIIA and; FIB and *aph(3)IA (aminoglycoside)*, and other resistance genes), biofilm strength and even virulence genes (*p* < 0.05). Of interest, rep FIB was significantly associated with a number of genes linked with antimicrobial resistance and heavy metals including copper (*pco*) and silver (*sil*) and quaternary ammonium compounds (*qac*) and integrons (*int*), suggesting most of these plasmids are likely resistance (R) plasmids.

### Antimicrobial and heavy metal resistance

**Figure 3:**
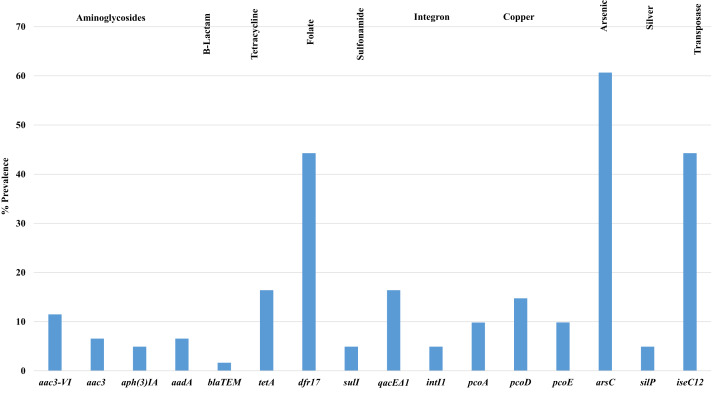
Prevalence of antimicrobial resistance genes detected in APEC examined. Antimicrobial resistance and heavy metal genes detected in APEC examined. Genes associated with tetracycline (*tetB)*, tellurite (*terB*, *terF*, *terX*, *terY3)*, silver (*silP)* and mercury (*merA)* were not detected in any isolate screened.

Figure 3 shows the prevalence of antimicrobial resistance and heavy metal genes detected among isolates examined, of note, there was a high prevalence of arsenical resistance (*arsC*—60.7%) and other metals including copper (*pco*) and silver (*sil*). Additional resistances observed included tetracycline (*tetA*) 16.4%; aminoglycosides (*aad*) 6.5%; beta-lactams (*bla*) 1.6%; gentamicin (*aac3*) 6.5%; and trimethoprim (*dfr17*) 44.3%. Additional genes associated with tetracycline (*tetB)*, tellurite (*terB*, *terF*, *terX*, *terY3)*, silver (*silP)* and mercury (*merA)* were not detected in any isolate screened.

Statistical analysis using the Mann–Whitney U test (Table S3D) for genotypic antimicrobial resistance and tissue of isolation, found significant associations (*p* < 0.05) between resistance genes such as *tetA*, *aph3IA*, *dfr 17*, *arsC* and *isec12* and *aac3VI* and tissues such as lungs vs bone marrow; lungs vs heart; bone marrow vs heart; heart vs ovary; heart vs yolk sac and lungs vs yolk sac. Additional comparisons using the chi-square test (Table S3E) for genotypic and phenotypic antimicrobial resistance among all APEC noted significant associations (*p* < 0.05) between antimicrobial resistance genes *aac3VIa/aac3VIb*, *aph3IA* and *tetA* and phenotypical resistance for gentamicin (GEN), tetracycline (TET), streptomycin (STR) and sulfisoxazole (FIS). In addition, significant relationships (*p* < 0.05) were also observed for plasmid replicon types and phenotypical resistance for strains harboring plasmids in particular I1 for (AUG, AMP, FOX, XNL and AXO); Y for NAL and TET; FIB for FIS; and FIA for AMP.

When plasmid replicons and antimicrobial resistance genes were assessed, significant relationships (*p* < 0.05) between the replicon type and antimicrobial resistance genes were noted (see above—plasmid replicons) including FIB and *aph(3)IA*, *intI1*, *aac3VI*, *sul* and *qacEΔ1*. In addition, these relationships also extend to heavy metals including copper, arsenic and silver. There also appears to be a significant association between biofilm strength and genotypic resistance such as *sul*, *qacEΔ1*, *aadA*, *aac3-VI*, in particular, strong biofilm is significantly associated with *aac3* (*p* < 0.05).

Phenotype analysis of all APEC isolates examined using the NARMS panel found approximately one third (17) of the isolates were resistant to 2 antimicrobial agents or less; another third (23) were resistant to 3 or more agents (Table 3) and the remainder (21 strains) were susceptible to all agents of the panel. Of note however, one strain (isolate #6) was found to be resistant to 9 different antimicrobials (Table 3).

**Table 3:** Antimicrobial resistance profiles among APEC examined.

Profile	Number of strains
Susceptible	21
TET	3
AMP, TET	4
AMP. GEN	1
GEN, TET	3
GEN, FIS	3
STR, TET	3
AMP, GEN, TET	5
GEN, STR, FIS	4
GEN, STR, TET	3
STR, FIS, TET	4
GEN, STR, FIS, TET	1
AUG, AMP, FOX, XNL, AXO	5
AUG, AMP, FOX, XNL, AXO, GEN, STR, FIS, TET	1

**Note:**

FOX, Cefoxitin; AZI, Azithromycin; CHL, Chloramphenicol; TET, Tetracycline; AXO, Ceftriaxone; AUG, Amoxicillin/clavulanic acid; CIP, Ciprofloxacin; GEN, Gentamicin; NAL, Nalidixic acid; XNL, Ceftiofur; FIS, Sulfisoxazole; SXT, Trimethoprim/sulfamethoxazole; AMP, Ampicillin; STR, Streptomycin.

Statistical analysis using the Mann–Whitney U test (Table S3D) noted significant associations between three phenotypes AMP, GEN and TET and strains isolated from organs including the lungs vs yolk sac; bone marrow vs yolk sac; ovary vs yolk sac; and TET for heart vs ovary; and GEN for liver vs yolk sac. When analysis for all phenotypes were compared, significant associations (*p* < 0.05) were observed between some resistance phenotypes (Table S3E) for example, GEN vs STR/FIS. Similarly, there were additional significant associations between phenotypes and resistance genotypes such as TET and *tetA*; FIS and STR and *qacEΔ1*. There were also significant associations between the resistance phenotype and plasmid replicons as described above.

### Biofilms

A total of 10 isolates (16.39%) classified as strong biofilm formers; 25 isolates (40.98%) as moderate; 23 isolates (37.7%) as weak and 3 isolates (4.91%) failed to form any biofilm among all isolates tested for biofilm forming ability (Table S1).

When examined overall, more than half of all isolates examined formed moderate to strong biofilms. Also of note, three strains failed to form any biofilm using minimal media. Biofilm density was weakly associated with the number of virulence genes harbored that is, the stronger the biofilm the greater number of virulence genes harbored. When analyzed for average number of virulence genes harbored, strong biofilms harbored an average of 12.3 genes compared to weak strains that harbored 6.56; while moderate biofilms harbored 9.48 genes. Of interest however, the three biofilm negligible strains harbored an average of 7.67 genes.

When statistical analysis using Tukey’s multiple comparisons (Table S3F) were used, no significant associations were found between tissue of isolation and biofilm strength and when biofilm types were compared for each individual tissue category examined. When compared across biofilm types (Table S3D), significant associations were noted for lung vs heart; bone marrow vs heart and heart vs yolk sac (moderate biofilms) and for heart vs yolk sac for weak biofilms (*p* < 0.05). Similarly, the chi-square test analysis for biofilm strength (Table S3E) found significant association (*p* < 0.05) across a range of characteristics including phenotypic resistance; fimbrial genes (*fim H*); iron related (*fyuA*); *iss*; *ompTp*; and antimicrobial resistance genes (e.g., *aph(3)IA*, *pcoA and pcoE*; *qacEΔ1*).

When biofilm type and antimicrobial resistance profiles were compared there did not appear to be a relationship between the biofilm type and antimicrobial resistance profile of the strain, as some strains with 3 or more resistances were weak biofilm formers and some moderate biofilm forming strains were susceptible to all agents of the NARMS panel strains; however, one strain (isolate #6) that was resistant to 9 antimicrobials was a strong biofilm former. Statistical analysis (chi-square) (Table S3E) did however identify a significant association between strength and phenotypic TET resistance (*p* < 0.05).

## Discussion

Serogroups of APEC detected in the current study were dominated by O78 (26.2%) followed by O2, O24, O25 and O86. Some of these serogroups have previously been reported in colibacillosis cases and are similar to other reports (Nolan et al., 2020; Rodriguez-Siek et al., 2005). However, given the diverse nature of APEC that can cause disease and even regional variation (Nolan et al., 2020), it is not unusual to see such diversity among disease causing APEC. Of particular interest in this study is the detection of serogroup O24 (prevalence of 16.3%), which has also been observed in other ongoing studies of this lab and we suspect is an emergent serogroup. Reasons as to why this serogroup appears to be emergent are currently unknown and will likely warrant further investigation. Similar work by Mir et al. (2019) assessing gut microflora of cattle vaccinated against and challenged with *E. coli* O157 reported shifts in the gut population associated with the second (booster) vaccination. Similarly, Gregersen et al. (2010) indicated that the Nobilis *E. coli* vaccine based on F11 also had an impact on the diversity of *E. coli* causing disease in broiler parent flocks likely affecting the population types present. The current study does not, however, have any information as to the vaccine status of the birds examined and can only speculate as to serogroups present or shifts in serogroups such as the detection of O24 and factors that could potentially influence its emergence. Regardless, further work on this serogroup is warranted.

A recent review of the literature failed to find any large reports on serogroup O24 but a study of 272 European APEC did identify O24 among a cluster of serogroups identified as MLST type ST117 with the cluster representing 14.7% of the APEC collection analyzed (Cordoni et al., 2016).

When multiple comparisons were assessed for serogroups detected in a tissue using Tukeys multiple comparison test no relationships were observed; however, when the tissue of isolation was compared for each individual serogroup, the only significant observations were for serogroup O24 which was significantly associated with bone marrow isolates. The role of O24 in bone marrow infection would suggest that this serogroup may be more invasive, however since little is known about this serogroup we can only speculate at this time and further investigation is warranted. Also of interest, most of the serogroups isolated in this study except for O24 and O186 have also been identified as shiga-toxin producing (STEC) strains according to Ludwig et al. (2020). However, we have not screened these strains for the presence of shiga-toxin genes and further investigation is warranted to confirm if these are STEC or harbor STEC-associated genes.

Phylogenetic analysis of the APEC examined in this study found most (52.3%) of the APEC classified in the B2 (24.5%) and F (27.8%) phylogenetic groups with the remaining isolates distributed over the A, B1 C, D and E phylogenetic groups. Data from this study is relatively similar to other analysis of phylogenetic groups reported by our research group (Logue et al., 2017) where most of the 452 APEC examined classified as B2 and F. In contrast, Barbieri et al. (2015) noted in their study of 52 APEC from Brazil, the majority of their collection classified as phylogenetic groups A and D with only 15% classifying as B2. Additionally, a study of 79 APEC from Korea (Kim et al., 2020) found that the majority of their collection (46.8%) was assigned to phylogenetic group D and only 7.6% was assigned to phylogenetic group B2 with the remainder of the collection assigned to A and B1 (22.8% each). However these assignments were made using the earlier Clermont classification scheme (Clermont, Bonacorsi & Bingen, 2000) and it would be interesting if application of the revised scheme would re-classify some of these strains. The current studies confirm our previous observations that the Clermont phylogenetic grouping scheme when applied to APEC do not all classify as the classic pathogenic type B2 as most extraintestinal strains of human origin do (Logue et al., 2017). Reasons for these differences are likely related to the genetic make-up of the strains examined and the potential for some of these strains to harbor additional pathogenicity islands which may influence virulence and overall classification (Logue et al., 2017).

When Tukeys multiple comparisons analysis was used to assess any potential association between phylogenetic grouping and tissue of isolation no significant differences were observed; similarly, when phylogenetic groups were compared for each tissue, no significant differences were found.

Additional analysis assessing the association or relationship between phylogenetic group and serogroup did find some significant relationships when the chi squared test was used. Of interest, significant differences were observed for phylogenetic B2 and serogroups O2 and O25, and F and serogroup O24 (*p* < 0.05). Some of these significant differences are based on a small number of isolates classifying in each group and therefore their importance should be viewed as limiting; however, the association of F with O24 does hold potential interest since phylogenetic group F has been shown to harbor additional pathogenicity island genes that contribute to APEC virulence (Logue et al., 2017).

Other analysis of serogroups and phylogenetic groups noted poor correlation between serogroups and phylogenetic groups. Literature has previously indicated that there is good correlations between phylogenetic groups and multi-locus sequence types (MLST) but not for serogroups (Cordoni et al., 2016). Moulin-Schouleur et al. (2007) noted clustering of *E. coli* strains of human and animal origin by phylogenetic group with some serogroups associated with the phylogenetic groups, however no single phylogenetic group was exclusively associated with a serogroup in the present study and may be a limitation of the size of the collection analyzed.

Virulence genes associated with APEC examined in this study varied with prevalence rates ranging from 5% to over 90% for some genes. Of interest, genes associated with the plasmid PAI, adhesins and toxins were found at a considerably high prevalence (>60% of isolates examined). In particular, genes associated with the APEC virulence plasmid ColV, (*iss, iroN, hlyF*, and *ompT*) were detected in 80–90% of strains examined suggesting that the ColV plasmid continues to have a defining role in APEC virulence. Work by others of our group (Johnson et al., 2006a) have also identified the ColV plasmid as a defining trait of the APEC pathotype (Johnson et al., 2006a, 2008a). Of interest however was the low prevalence of the *cvaC* gene which is part of the ColV structural operon associated with colicin V a polypeptide toxin (bacteriocin) that is produced against other *E. coli* and closely related bacteria. Previous work has detected *cvaC* at a prevalence of 67% (Rodriguez-Siek et al., 2005) in a collection of over 500 APEC from poultry while a later study of APEC from turkey cellulitis found the prevalence of *cvaC* to be <10% in cellulitis strains and not detected in systemic isolates (De Oliveira et al., 2020). This disparity may be related to some of the strains examined being more related to ColBM type plasmids than ColV their likely ancestor or there is rearrangement/deletion of portions of the ColV operon (Johnson, Johnson & Nolan, 2006; Johnson et al., 2006a). Further sequencing is warranted to identify the types of Col plasmids these strains harbor. Other virulence traits of APEC included in this analysis such as adhesins found prevalence of *fimH* and *ompT* (25–65% prevalence) were high compared to other genes such as *pap*, *foc* and *sfaS* but were relatively similar to rates observed in cellulitis isolates (De Oliveira et al., 2020) and in APEC (Rodriguez-Siek et al., 2005).

When compared with biofilm forming capabilities, all of the strong biofilm forming strains harbored genes associated with the ColV plasmid; however, isolates that classified as moderate and weak biofilm formers, also appeared to harbor the ColV plasmid while a third group of isolates that did not harbor ColV traits and still formed biofilm suggesting that the presence of the ColV plasmid and biofilm formation are probably not linked.

Also of interest in the current study was the assessment of antimicrobial susceptibility and antimicrobial resistance gene possession. A considerable number (34%) of isolates screened were susceptible to all antimicrobial agents of the NARMS panel; while 54% of strains were resistant to 3 drugs or less and the remaining strains (9.7%; 6 strains) were resistant to 4 and 5 drugs and one single strain was resistant to 9 antimicrobials. These data differ from other APEC reports of our group (Johnson et al., 2012a) where the prevalence of multidrug resistant APEC were considerably greater in strains assessed and most had 8 resistance phenotypes or less. Similarly, a 2012 survey of 144 APEC from severe cellulitis lesions in Brazil noted that 82% of their isolates examined were phenotypically resistant to 1–5 antimicrobials (Barbieri et al., 2013). While many other reports exist worldwide reporting multiple resistances of interest, Subedi et al. (2018) reported 94% of APEC (47 of 50) examined in Nepal were resistant to 3 or more drugs, and studies in Italy of 48 APEC (Piccirillo et al., 2014) and 331 APEC from Canada (Varga et al., 2018) noted 94% and 46% respectively were resistant to 3 or more classes of antimicrobials; and 77% of 116 APEC in Egypt (Awad, Arafat & Elhadidy, 2016) were classified as multidrug resistant.

While comparisons of antimicrobial resistance in APEC across the nation and globally differ, what is evident is that the small collection of isolates examined in this study do appear to have a lower prevalence of resistance and multi-drug resistance. These APEC come from a region where approaches such as raising birds antibiotic free and no antibiotics ever in production are commonplace. In addition, these isolates were collected post implementation of the veterinary feed directive (VFD) (Food and Drug Administration, 2015) which may also have an influence on the results observed. Our speculations are further supported by a recent report by Singer & Porter (2019) and Singer et al. (2020a, 2020b) who surveyed chicken and turkey producers nationally (2013–2017) and found significant reductions in the on-farm use of antimicrobials in feed and at the hatchery. Similarly, the overall low prevalence of plasmid replicons detected in our isolates further supports our observations on the phenotypical resistance prevalence. Although detailed information as to the sources of strains and operations from where these isolates originated is limited, a larger study of farm types (and practices) and sources of disease strains is warranted to better understand the potential impact of the VFD and alternative farming practices on the antimicrobial resistance of APEC. Also of importance was the types of resistance observed which included tetracycline, the aminoglycosides (gentamicin, streptomycin), penicillins (ampicillin), the folate pathway inhibitors (sulfisoxazole), and the cephalosporins (cefoxitin and ceftiofur). Similar such resistances have been observed in APEC of our lab and most often these resistances may reflect natural resistances or as a result of previous prescribing practices and the persistence of these resistances as a result of genes associated with these traits. Some of the resistances observed in our study were confirmed as linked with gene presence which was evident for the aminoglycodsides (*aac*, *aad* and *aph* genes), tetracyclines (*tetA*) and the folate pathway inhibitors (*dfr17*).

Biofilm formation was a significant activity of most APEC examined. A study examining *E. coli* recovered from chicken meat and wildlife poultry to assess the significance of biofilm found only 68% of isolates were biofilm formers (Pavlickova et al., 2017), but in the current study 95% of the isolates examined formed some type of biofilm. Differences observed in biofilm strength is likely due to a number of factors including the types of poultry isolates examined, the disease state of the birds and even the media used for biofilm analysis. In the current study, all isolates were recovered from lesions of colibacillosis, while a comparative study by Pavlickova et al. (2017) sourced isolates recovered from skin tissue of the legs and breast of chicken carcasses from a Czech market supplier and isolates from the feces of wild birds. Isolates used in their study are more likely to originate from healthy birds (since they were available for retail) and are more likely to be commensal (fecal strains) contaminating the carcass.

Similar work by Skyberg et al. (2006, 2007) noted better biofilm among commensal (fecal) strains than APEC, but the influence of media type affecting biofilm strength was also noted. The Pavlickova et al. (2017) study determined biofilm in trypticase soy broth—a relatively nutrient rich medium which may also account for some differences observed. In a second study recently reported by Nielsen et al. (2018), 56.8% of the APEC isolates examined produced negligible biofilms with 43.2% of the APEC examined producing some level of biofilm; this data contrasts sharply with that of the current study where 95% of strains were biofilm formers with the majority of strains producing moderate to weak biofilms and only three isolates were non-biofilm formers. The APEC isolates of the Nielsen et al. (2018) were from a collection where some of the isolates were more than 20 years old; while those of the current study were collected more recently (Aug–Dec 2018) and may better reflect the types of APEC currently circulating in poultry flocks causing disease.

## Conclusion

In conclusion, this study characterized APEC from various tissues of diagnostic cases of colibacillosis. A significant number of strains examined harbored traits of the ColV plasmid—a defining trait of APEC. However, possession of the ColV did not appear to influence or be linked with biofilm formation. APEC that are currently circulating in production poultry would appear to be well defined pathogens with the capabilities for harboring virulence and resistance traits and producing biofilms. Of significant interest was the level of phenotypic antimicrobial resistance of the APEC from different tissues which was relatively low and it would appear that the current approaches associated with management (no antibiotics) and the implementation of the VFD would appear to be impacting resistance observed. Further work to assess virulence in relation to tissue of origin of APEC may shed light on virulence traits of organ specific strains and their potential role in the infection/disease process.

## Supplemental Information

10.7717/peerj.11025/supp-1 Supplemental Information 1 Raw data for all isolates of APEC examined.

10.7717/peerj.11025/supp-2 Supplemental Information 2 Primers Used. Primers used for the amplification of gene/traits of interest.

10.7717/peerj.11025/supp-3 Supplemental Information 3 Statistical analysis of all isolates examined. Statistical analysis of all isolates for characteristics such as serogroup, phylogenetic type, tissue of isolation, virulence genes, antimicrobial resistance, genetic resistance, and biofilm type.

## Additional Information and Declarations

### Data Availability

The following information was supplied regarding data availability: All data is available in the Supplemental Files.
