# Supplementary material for: Characterizing avian pathogenic Escherichia coli (APEC) from colibacillosis cases, 2018
Source: PeerJ. 2021 Mar 4;9:e11025. doi: 10.7717/peerj.11025 (PMC7937341; doi:10.7717/peerj.11025)
Supplement: Supplemental Information 2 — Primers used for the amplification of gene/traits of interest. [file peerj-09-11025-s002.docx]

**Supplementary Table 2: Primers used for the amplification of gene/traits of interest.**

***Gene/trait Amplicon Primer sequence (5’ – 3’) Reference/ comments***

***size (bp)***

***16S***

UAL 1939b 187 F ATGGAATTTCGCCGATTTTGC

UAL 2105b R ATTGTTTGCCTCCCTGCTGC (Lamprecht et al. 2014)

***Plasmid PAI encoded traits***

*cvaC** 679 F cacacacaaacgggagctgtt (Johnson & Stell 2000)

R cttcccgcagcatagttccat

*iroN*† 667 F aagtcaaagcaggggttgcccg

R gacgccgacattaagacgcag (Rodriguez-Siek et al. 2005)

*traT** 290 F ggtgtggtgcgatgagcacag (Johnson & Stell 2000)

R cacggttcagccatccctgag

*iss* 323 F CAGCAACCCGAACCACTTGATG (Johnson et al. 2008)

R agcattgccagagcggcagaa

*ompTp* 496 F TCATCCCGGAAGCCTCCCTCACTACTAT Plasmid-encoded

R TAGCGTTTGCTGCACTGGCTTCTGATAC outer membrane protease

*iutA(aerJ)* 302 F ggctggacatcatgggaactgg (Johnson & Stell 2000)

R cgtcgggaacgggtagaatcg

***Chromosmal encoded traits***

***Adhesins***

*bmaE* 507 F atggcgctaacttgccatgctg (Johnson & Stell 2000) R agggggacatatagcccccttc

*fimH* 508 F tcgagaacggataagccgtgg (Johnson & Stell 2000)

R gcagtcacctgccctccggta

*focG* 364 F cagcacaggcagtggatacga (Johnson & Stell 2000)

R gaatgtcgcctgcccattgct

*gafD* 952 F tgttggaccgtctcagggctc (Johnson & Stell 2000)

R tcccggaactcgctgttact

*papA* 717 F atggcagtggtgttttggtg (Johnson & Stell 2000)

R cgtcccaccatacgtgctcttc

*papC* 205 F gtggcagtatgagtaatgaccgtta (Johnson & Stell 2000)

R atatcctttctgcagggatgcaata

*papEF* 326 F gcaacagcaacgctggttgcatcat (Johnson & Stell 2000)

R agagagagccactcttatacggaca

*papG* allele I§ 461 F tcgtgctcaggtccggaattt (Johnson & Stell 2000)

R tggcatcccccaacattatcg

*papG* allele I’§ 479 F ctactatagttcatgctcaggtc (Johnson & Stell 2000)

R cctgcatcctccaccattatcga

*papG* allele II§ 190 F gggatgagcgggcctttgat (Johnson & Stell 2000)

R cgggcccccaagtaactcg

*papG* allele III§ 258 F ggcctgcaatggatttacctgg (Johnson & Stell 2000)

R ccaccaaatgaccatgccagac

*papG1** 1140 F CTGTAATTACGGAAGTGATTTCTG

R TTCCAGAAATAGCTCATGTAACCCG

*papG2/3* 1070 R ACTATCCGGCTCCGGATAAACCAT

*sfaS* 244 F gtggatacgacgattactgtg (Johnson & Stell 2000)

R ccgccagcattccctgtattc

*sfa-foc* 410 F ctccggagaactgggtgcatcttac (Johnson & Stell 2000)

R cggaggagtaattacaaacctggca

*adhC* 411 F GTCGGCGAGGGCGTCACCAGCCTGAAGC (Johnson et al. 2007a)

R GGCCGCGCCCTGAATCACCGCCAGACC

*ompT* 559 F atctagccgaagaaggaggc

R cccgggtcatagtgttcatc (Rodriguez-Siek et al. 2005)

*fliC* (H7) 547 F acgatgcaggcaacttgacg

R gggttggtcgttgcagaacc (Rodriguez-Siek et al. 2005)

***Iron related***

*fyuA* 787 F tgattaaccccgcgacgggaa (Johnson & Stell 2000)

R cgcagtaggcacgatgttgta

*ireA* 254 F gatgactcagccacgggtaa

R ccaggactcacctcacgaat (Rodriguez-Siek et al. 2005)

*etsB* 537 F CAGCAGCGCTTCGGACAAAATCTCCT *E. coli* transport system

R TTCCCCACCACTCTCCGTTCTCAAAC

iroN_ec_ 667 F AAGTCAAAGCAGGGGTTGCCCG Salmochelin operon

R GACGCCGACATTAAGACGCAG

***Protectins***

*kpsMT K1* 153 F TAGCAAACGTTCTATTGGTGC (Johnson & Stell 2000)

R catccagacgataagcatgagca

*kpsMT* *II* 272 F gcgcatttgctgatactgttg (Johnson & Stell 2000)

R catccagacgataagcatgagca

*kpsMT* *III* 392 F tcctcttgctactattccccct (Johnson & Stell 2000)

R aggcgtatccatccctcctaac

***Toxins***

*cdtB* 430 F GAAAATAAATGGAACACACATGTCCG (Johnson & Stell 2000) F’ GAAAGTAAATGGAATATAAATGTCCG

R AAATCTCCTGCAATCATCCAGTTA

R’ AAATCACCAAGAATCATCCAGTTA

*cnf1* 1105 F ATCTTATACTGGATGGGATCATCTTGG (Johnson & Stell 2000)

R GCAGAACGACGTTCTTCATAAGTATC

*hlyD* 904 F ctccggtacgtgaaaaggac

R gccctgattactgaagcctg (Rodriguez-Siek et al. 2005)

*hlyF* 599 F GGCGATTTAGGCATTCCGATACTC Hemolysin F

R ACGGGGTCGCTAGTTAAGGAG

***Miscellaneous***

*ibeA* 171 F AGGCAGGTGTGCGCCGCGTAC (Johnson & Stell 2000)

R TGGTGCTCCGGCAAACCATGC

*maIX PAI* 925 F GGACATCCTGTTACAGCGCGCA (Johnson & Stell 2000)

R TCGCCACCAATCACAGCCGAAC

*1024UI*  598 F GGCTTTCCCGCCTTCTTTTACCACTACG Inc FIB

R GGACGGCGACGTTGTGTTATTCGGTAAT recombinase/integrase

*1051UI* 664 F CACGCCGTTACTGGTCGCGGAAAAAT ColV gene

R AACCCACGGCCTCTATTGGCGAAGAACT

*parB*  534 F TCGTGGCCGAGTTCTTGGCAACAGC Inc FIB

R GCGGCCTGAAACGCACGAGTCACTTT Plasmid partitioning

*umuC*  474 F CCGCCGTACGGAAAAACTGCTGTCACTG UV protection gene R ACGGCAGCGGCAATGATGTCCTGTGTAT

*rfc* 788 F ATCCATCAGGAGGGGACTGGA O antigen polymerase

R AACCATACCAACCAATGCGAG

*iha* 829 F CTGGCGGAGGCTCTGAGATCA UPEC Island

R TCCTTAAGCTCCCGCGGCTGA

*afa* 594 F GGCAGAGGGCCGGCAACAGGC Afimbrial adhesin Afa

R CCCGTAACGCGCCAGCATCTC

**Plasmid-Replicon Types**

B/O 159 F GCGGTCCGGAAAGCCAGAAAAC

R TCTGCGTTCCGCCAAGTTCGA (Johnson et al. 2007b)

FIC 262 F GTGAACTGGCAGATGAGGAAGG

R TTCTCCTCGTCGCCAAACTAGAT (Johnson et al. 2007b)

A/C 465 F GAGAACCAAAGACAAAGACCTGGA

R ACGACAAACCTGAATTGCCTCCTT (Johnson et al. 2007b)

P 534 F CTATGGCCCTGCAAACGCGCCAGAAA

R TCACGCGCCAGGGCGCAGCC (Johnson et al. 2007b)

T 750 F TTGGCCTGTTTGTGCCTAAACCAT

R CGTTGATTACACTTAGCTTTGGAC (Johnson et al. 2007b)

K/B 160 F GCGGTCCGGAAAGCCAGAAAAC

R TCTTTCACGAGCCCGCCAAA (Johnson et al. 2007b)

W 242 F CCTAAGAACAACAAAGCCCCCG

R GGTGCGCGGCATAGAACCGT (Johnson et al. 2007b)

FIIA 270 F CTGTCGTAAGCTGATGGC

R CTCTGCCACAAACTTCAGC (Johnson et al. 2007b)

FIA 462 F CCATGCTGGTTCTAGAGAAGGTG

R GTATATCCTTACTGGCTTCCGCAG (Johnson et al. 2007b)

FIB 702 F GGAGTTCTGACACACGATTTTCTG

R CTCCCGTCGCTTCAGGGCATT (Johnson et al. 2007b)

Y 765 F AATTCAAACAACACTGTGCAGCCTG

R GCGAGAATGGACGATTACAAAACTTT (Johnson et al. 2007b)

I1 139 F CGAAAGCCGGACGGCAGAA

R TCGTCGTTCCGCCAAGTTCGT (Johnson et al. 2007b)

X 376 F AACCTTAGAGGCTATTTAAGTTGCTGAT

R TGAGAGTCAATTTTTATCTCATGTTTTAGC (Johnson et al. 2007b)

HI1 471 F GGAGCGATGGATTACTTCAGTAC

R TGCCGTTTCACCTCGTGAGTA (Johnson et al. 2007b)

N 559 F GTCTAACGAGCTTACCGAAG

R GTTTCAACTCTGCCAAGTTC (Johnson et al. 2007b)

HIII 644 F TTTCTCCTGAGTCACCTGTTAACAC

R GGCTCACTACCGTTGTCATCCT (Johnson et al. 2007b)

L/M 785 F GGATGAAAACTATCAGCATCTGAAG

R CTGCAGGGGCGATTCTTTAGG (Johnson et al. 2007b)

**Antimicrobials and Heavy Metals**

*blaTEM* 558 F ATGTGCGCGGAACCCCTATTTGTTTA Ampicillin resistance

R AAAAAGCGGTTAGCTCCTTCGGTCCT

*aac3 VIa* 502 F GGCACCCGCGACGCCCTGGTCCAAAAG Gentamicin resistance

R GGGCCCGGCGCCGATCGACAGGATTT

*aac3 VIb* 302 F GGGCAAGCGCCGCGTCACTTATT Gentamicin resistance

R CGCGGCGTTGTTTCGGCTTCA

*tetA*  372 F CGGGGCGACTGGGGCGGTAGC Tetracycline resistance

R CAAAGCGCGGCCGGCACCTGT

*tetB* 446 F AACGCGTGAAGTGGTTCGGTTGGT Tetracycline resistance

R TTCGCCCCATTTAGTGGCTATTCTTC

*aph3IA*  378 F TCGGGCAATCAGGTGCGACAATCTA Gentamicin resistance

R TGCCAGCGCATCAACAATATTTTCACC

*terD* 231 F CCACTGCGCGGAATTTCCACTCACCAT Tellurite resistance

R ACGCCGTCCCGTCTGATGTTGACAAG

*terF*  428 F CCGACAAACTTCCAGAAGATGGGGTAGT Tellurite resistance

R GAGGCAGCGGTTGCATTTGTACTTGACG

*terX*  576 F ATGCGCCGCCTGCCTGTTTACCTTGTTA Tellurite resistance

R CGCGCTTGTGCTGCCGGAAGACA

*terY3* 302 F CCTGGGGCCGTCAGCGGACCTG Tellurite resistance

R TCCTTGCTGGTGGCCGTTCATACTTCAT

*pcoA*  507 F ATCCGGAAGGTCAGCACCGTCCATAGAC Copper resistance

R GACCTCGCGGATGTCAGTGGCTACACCT

*pcoD*  502 F GGCGCCCAGAATGATAATCGCAACA Copper resistance

R GGGCGTGGCGCTGGCTACACTT

*pcoE* 385 F GTGGGGCAGCTTTTGCTCAGTCCAGTGA Copper resistance

R CGAAGCTTTCTTGCCTGCGTCTGATGTG

*dfr17*  243 F ATATCCCGTGGTCAGTAAAAGGTG Trimethoprim resistance

R GACCCCCGCCAGAGACATA

*arsC* 153 F CCAGCCTGCGGCACCTCGCGTAATAC Arsenic resistance

R ACGCAGCAGCGCTCGTACTGAAATACCC

*silE*  364 F TCGGCCTGGGCCACTGAAACCGTGAATA Silver resistance

R GGCGGTGCGCTTCGGCCATAGCCTGATG

*silP* 603 F ACACCCCGGCCTGGGCTCCTT Silver resistance

R TGCGGGCACGGGAACAAACCTC

*sul1* 462 F CGCCGCTCTTAGACGCCCTGTCC Sulfa resistance

R CAACGGTGGCGCCCAAGAAGGAT

*merA* 250 F GATCCGCGCCGCCCATATCGCCCATCTG Mercury resistance

R CACGCGCTCGCCGCCGTCGTTGAGTTG

*intI1* 545 F CACTCCGGCACCGCCAACTTTC Integrase

R GAACGGGCATGCGGATCAGTGAG

*iseC12* 404 F CGCGGCCACGTAAACCGAAAGATAAA Transposase

R GCGCGGGTGCACAGCAACCTC

*aadA*  365 F TAACGGCGCAGTGGCGGTTTTCA Aminoglycoside

R AAGCTCGCCGCGTTGTTTCATCAAG resistance

*qacE delta* 250 F TCGGCCTCCGCAGCGACTTCC Quaternary

F CTTGCCCCTTCCGCCGTTGTCTAAT ammonium compounds

**Phylogenetic Typing**

*chuA* 288 F ATGGTACCGGACGAACCAAC (Clermont et al. 2013)

R TGCCGCCAGTACCAAAGACA (Clermont et al. 2000)

*yjaA* 211 F CAAACGTGAAGTGTCAGGAG (Clermont et al. 2013)

R AATGCGTTCCTCAACCTGTG Quadruplex PCR

TspE4.C2 152 F CACTATTCGTAAGGTCATCC (Clermont et al. 2013)

R AGTTTATCGCTGCGGGTCGC

*arpA* 400 F AACGCTATTCGCCAGCTTGC (Clermont et al. 2013)

R TCTCCCCATACCGTACGCTA (Clermont et al. 2004)

*arpA** 301 F GATTCCATCTTGTCAAAATATGCC (Lescat et al. 2013) R GAAAAGAAAAAGAATTCCCAAGAG Group E

*trpA* 219 F AGTTTTATGCCCAGTGCGAG (Lescat et al. 2013)

R TCTGCGCCGGTCACGCCC Group C

Clermont O, Bonacorsi S, and Bingen E. 2000. Rapid and simple determination of the Escherichia coli phylogenetic group. *ApplEnvironMicrobiol* 66:4555-4558.

Clermont O, Bonacorsi S, and Bingen E. 2004. Characterization of an anonymous molecular marker strongly linked to Escherichia coli strains causing neonatal meningitis. *J Clin Microbiol* 42:1770-1772. 10.1128/jcm.42.4.1770-1772.2004

Clermont O, Christenson JK, Denamur E, and Gordon DM. 2013. The Clermont Escherichia coli phylo-typing method revisited: improvement of specificity and detection of new phylo-groups. *Environ Microbiol Rep* 5:58-65. 10.1111/1758-2229.12019

Johnson JR, and Stell AL. 2000. Extended virulence genotypes of *Escherichia coli* strains from patients with urosepsis in relation to phylogeny and host compromise. *J Infect Dis* 181:261-272. JID990990 [pii] 10.1086/315217

Johnson TJ, Kariyawasam S, Wannemuehler Y, Mangiamele P, Johnson SJ, Doetkott C, Skyberg JA, Lynne AM, Johnson JR, and Nolan LK. 2007a. The Genome Sequence of Avian Pathogenic *Escherichia coli* Strain O1:K1:H7 Shares Strong Similarities with Human Extraintestinal Pathogenic *E. coli* Genomes. *Journal of Bacteriology* 189:3228-3236. JB.01726-06 [pii] 10.1128/JB.01726-06

Johnson TJ, Wannemuehler YM, Johnson SJ, Logue CM, White DG, Doetkott C, and Nolan LK. 2007b. Plasmid Replicon Typing of Commensal and Pathogenic *Escherichia coli* Isolates. *Applied and Environmental Microbiology* 73:1976-1983. AEM.02171-06 [pii] 10.1128/AEM.02171-06

Johnson TJ, Wannemuehler YM, and Nolan LK. 2008. Evolution of the *iss* gene in *Escherichia coli*. *Appl Environ Microbiol* 74:2360-2369. AEM.02634-07 [pii] 10.1128/AEM.02634-07

Lamprecht C, Romanis M, Huisamen N, Carinus A, Schoeman N, Sigge GO, and Britz TJ. 2014. Escherichia coli with virulence factors and multidrug resistance in the Plankenburg River. *South African Journal of Science* 110:32-37. ARTN 2013-0347 10.1590/sajs.2014/20130347

Lescat M, Clermont O, Woerther PL, Glodt J, Dion S, Skurnik D, Djossou F, Dupont C, Perroz G, Picard B, Catzeflis F, Andremont A, and Denamur E. 2013. Commensal Escherichia coli strains in Guiana reveal a high genetic diversity with host-dependant population structure. *Environ Microbiol Rep* 5:49-57. 10.1111/j.1758-2229.2012.00374.x

Rodriguez-Siek KE, Giddings CW, Doetkott C, Johnson TJ, Fakhr MK, and Nolan LK. 2005. Comparison of *Escherichia coli* Isolates Implicated in Human Urinary Tract Infection and Avian Colibacillosis. *Microbiology* 151:2097-2110.
